# Contemporary Theory and Research

**Published:** 1893-09-09

**Authors:** 


### Contemporary Theory and Research.

### THE ZOOTHERMIC INSTITUTE AT ROME.

In a contemporary M. Pion gives a description more curious than attractive of a method of treatment which reads like a survival from barbaric times. He writes: "French doctors will not be sorry to have a recent description of the famous Zoothermic Institute, which provides in perfect style a certain mode of healing much esteemed among Italians. In the abattoirs, fitted up in the most luxurious style, after the latest fashions, and finished only last year, is a special building which I was much surprised to find there. To this place, under the best management, doctors send their patients tortured by rheumatism and gout. Others, anæmic patients, prostrated by the malaric fevers of the Pontine marshes, come there in order to drink blood. The unhappy invalids, without any repulsion, dip their arms, legs, or whole bodies in the warm fluid. The municipality has paid all the expenses of the installation. The prices vary from 3 francs to 75 centimes, while for the extreme poor the treatment is free. There is room for more than fifty patients at a time, and the veterinary inspectors assured me that the results are appreciably beneficial. In a hall above glasses are prepared into which pure blood is poured freed from fibrine and above suspicion. The animals are previously inspected by the veterinary official. The blood is freed from fibrine by being placed in a kind of round box, hermetically sealed, in which turns a bar of iron, terminated by three teeth. The apparatus revolves after the fashion of an ordinary coffee mill, and the process being performed under cover from the air no microbe or dangerous germ can penetrate into the liquid."

### HARDY SPORES.

A writer in *Nature* quotes an interesting example of the degree of resistance to high temperatures exhibited by some micro-organisms, and published in the *Centralblatt für Bacteriologie*. Whilst preparing nutritive gelatin-peptone in the usual manner, Heine found that, despite all precautions of sterilisation, &c., numerous yellow or reddish-yellow centres subsequently appeared in the culture material. On isolating out these colonies and further studying them, the growths were ascertained to be derived from two spore-producing bacilli, which had resisted the usual 10 to 20 minutes' exposure to steam on three successive days. On further studying these organisms, it was found that one of them required three hours' continuous steaming before being destroyed, whilst the other was not annihilated until this had been prolonged for seven hours. Still more recently a cladothrix has been found in water which, on account of its ability to resist high temperatures, has been called cladothrix invulnerabilis. It was still endowed with vitality after having undergone six successive exposures to ordinary intermittent sterilisation at 100 deg. C.

### CHOLERA AND THE WEATHER.

Dr. Dignat, in a valuable series of observations, seeks to establish under what meteorological conditions cholera and influenza are most favourably developed. He inclines to think that no one condition can be found to exercise by itself a preponderating influence in epidemics of this nature, but certain phenomenal combinations he finds present in all outbreaks which he has had the opportunity of studying from this point of view. His researches cannot, of course, extend back many years, since systematic meteorological observations have not till recently been obtainable. He gives the following *résumé* of his conclusions: "(1.) The development of epidemics of influenza and of epidemics of cholera seem to be favoured by certain meteorological circumstances, invariably to be found at the moment of their appearance. (2.) These circumstances are almost exactly the same, whether it is a question of cholera or simply of influenza. (3.) They are determined: (*a*) By the dominant direction of the east, north-east, or north winds during the days preceding the epidemic; (*b*) by the lowering towards the same period and during the whole duration of the epidemic of the electric influence below the normal; (*c*) by marked variations, sometimes more, sometimes less of the temperature, above the normal average temperature of the corresponding period; (*d*) by similar variations of barometric pressure, these variations consisting always rather in an augmentation of pressure; (*e*) finally, and above all, by the coincidence of an elevation of this pressure with the lowering of the electric influence. M. Dignat considers it the greatest error to attribute to variations in the rainfall any influence whatever on outbreaks of influenza or cholera.

